# 

**Published:** 1887-09

**Authors:** 


					﻿hay66ahkahm aja ka aiiio aiia oao aioio
Vol. III
SEPTEMBER. 1887
No. 3.
DANIEL'S
MEDICAL
JOURNAL
F. E. DANIEL., hahjaakkhja hanajhn354 ja64%$# 
^&Hayha7$#gbv^$%@gha aj
GGTaghbayah^%$ga a* %%$ HGFWW*
				

